# PD-L1 Expression in NSCLC: Clouds in a Bright Sky

**DOI:** 10.3390/ijms26136066

**Published:** 2025-06-24

**Authors:** Victoria Ferrari, Jocelyn Gal, Baharia Mograbi, Gerard Milano

**Affiliations:** 1 Antoine Lacassagne Center, Department of Medical Oncology, University Côte d’Azur, 33 Avenue de Valombrose, 06189 Nice, France; victoria.ferrari@nice.unicancer.fr; 2 Antoine Lacassagne Center, Department of Epidemiology and Biostatistics, University Côte d’Azur, 33 Avenue de Valombrose, 06189 Nice, France; jocelyn.gal@nice.unicancer.fr; 3 FHU OncoAge, IHU RespirERA, IRCAN, Inserm, CNRS 7284, U1081, University Côte d’Azur, 06189 Nice, France; baharia.mograbi@univ-cotedazur.fr; 4 Antoine Lacassagne Center, University Côte d’Azur, 33 Avenue de Valombrose, 06189 Nice, France

**Keywords:** immunotherapy, non-small cell lung cancer, PD-L1, gene expression

## Abstract

Programmed Death-Ligand 1 (PD-L1) is a major target for immunotherapy using checkpoint inhibitors (CPIs), particularly in lung cancer treatment. Tumoral PD-L1 expression has been recognized as a natural predictor of CPI response. This predictive relationship is primarily due to its upregulation by interferon-gamma, which is released by immune cells (mainly T lymphocytes and natural killer cells) in proximity to tumor cells, driving an immune resistance mechanism. However, PD-L1 expression is modulated at multiple levels, including oncogenic signaling pathways, and transcriptional and post-transcriptional regulations, potentially leading to false positive predictions. Conversely, variable glycosylation of PD-L1 may compromise the accuracy of immunohistochemical measurements, resulting in false negative predictive data. In addition, PD-L1 expression demonstrates relative instability throughout treatment courses (e.g., chemotherapy and tyrosine kinase inhibitors), further limiting its clinical utility. In this review, we focused on the molecular mechanisms governing PD-L1 expression with a special emphasis on lung cancer. We also discussed biomarker strategies for optimizing patient selection for checkpoint inhibitor therapy where multimodal/multi-omics meta-biomarker approaches are emerging. Such comprehensive PD-L1-enriched biomarker strategies require evaluation through large-scale prospective studies, particularly in lung cancer, where numerous competing predictive candidates exist for CPI response.

## 1. Introduction

### The PD-1/PD-L1 Axis in Immune Regulation and Cancer Therapy

Programmed death-ligand 1 (PD-L1) represents a critical immunoregulatory molecule that shapes adaptive immune responses. Widely recognized for its immunosuppressive functions, PD-L1 serves as a pivotal negative regulator of T cell-mediated immunity [1]. PD-L1 is a checkpoint protein expressed across diverse cell types, including dendritic cells and lymphocytes. Notably, tumor cells frequently upregulate PD-L1 expression to evade killing by cytotoxic T lymphocytes [2]. The biological activity of PD-L1 is primarily mediated through its interaction with programmed death-1 (PD-1), a receptor predominantly expressed on activated CD8+ cytotoxic T lymphocytes. Upon PD-L1 binding, PD-1 triggers an inhibitory signaling cascade that attenuates T cell receptor (TCR) signaling, repressing the proliferation and cytolytic function of these cells [3]. In the tumor microenvironment, the PD-1/PD-L1 pathway functions as a critical “immune brake” that contributes to tumor immune evasion.

PD-L1 represents a major target of immunotherapy, which aims at reactivating the immune system against cancer cells. Therapeutic monoclonal antibodies targeting either PD-1 (nivolumab, pembrolizumab) or PD-L1 (atezolizumab, durvalumab, and avelumab) constitute the core of immune checkpoint inhibitors and have transformed the treatment landscape for numerous malignancies. These biologic agents function by disrupting the PD-1/PD-L1 interaction, thereby releasing the inhibitory brake on T cell function and reinvigorating exhausted tumor-specific lymphocytes. This therapeutic restoration of anti-tumor immunity has yielded unprecedented clinical responses, including durable complete remissions in previously refractory cancers [4].

The revolutionary impact of checkpoint inhibition is perhaps most evident in non-small cell lung cancer (NSCLC), where immunotherapy has dramatically altered the natural history of this historically treatment-resistant malignancy. Prior to the advent of immunotherapy, patients with advanced NSCLC faced dismal five-year survival rates of approximately 5% [5]. However, contemporary data from long-term follow-up studies demonstrate that a subset of patients receiving PD-1/PD-L1 inhibitors achieve sustained clinical responses extending beyond five years, an outcome previously inconceivable with conventional cytotoxic chemotherapy regimens [6].

This position paper aims to provide a comprehensive analysis of the molecular mechanisms governing PD-L1 expression and to discuss emerging biomarker strategies for optimizing patient selection for checkpoint inhibitor therapy, with a particular emphasis on NSCLC. By integrating PD-L1 into composite predictive algorithms, one could make clear the cloudy limitations of PD-L1 IHC (immunohistochemistry) alone and develop more nuanced approaches to treatment stratification.

## 2. Mechanisms and Factors Influencing PD-L1 Expression

### 2.1. Mechanisms of PD-L1 Expression Regulation

Interferon-gamma (IFN-γ), primarily produced by T cells and natural killer (NK) cells, is a major inducer of PD-L1 expression through the JAK-STAT-IRF1 intracellular signaling pathway. Elevated PD-L1 expression in tumors can thus indicate a high presence of T cells in the tumor microenvironment, creating conditions for tumor-induced immunosuppression [7]. Given this immunological interplay, measuring tumor PD-L1 expression has been considered an ideal predictive biomarker for checkpoint inhibitor efficacy.

**Figure 1:**
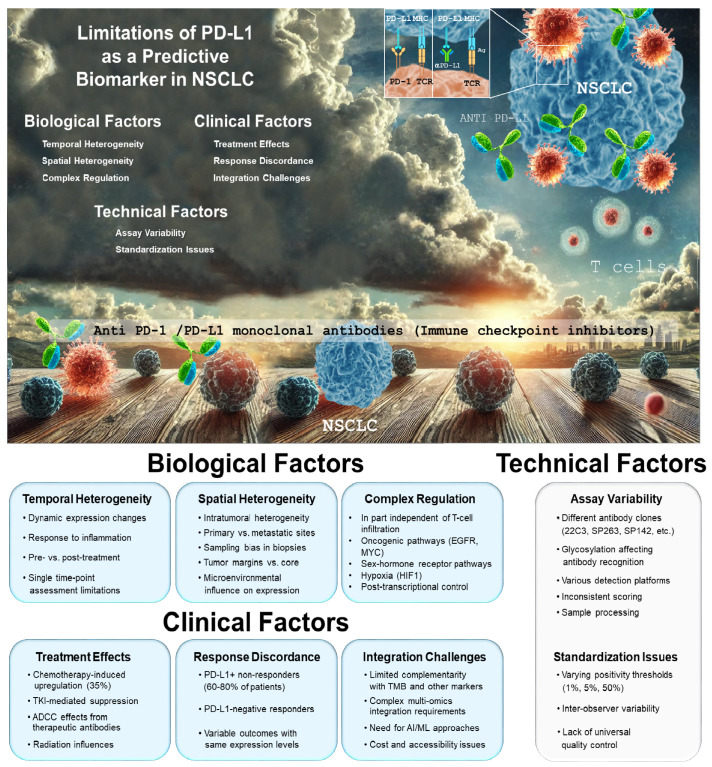
PD-L1 expression in NSCLC: clouds in a bright sky.

However, the regulation of PD-L1 expression extends beyond IFN-γ-mediated immune signaling [8]. PD-L1 expression can be modulated at multiple levels, including the initiation of gene transcription, post-transcriptional regulation, and post-translational protein modifications [2,9,10]. Oncogenic signaling pathways, such as MYC (myelocytomatosis viral oncogene) and EGFR (epidermal growth-factor receptor), activate transcription factors that upregulate PD-L1 expression [11]. Of note, sex hormone receptors exert a negative influence on PD-L1 expression. Hypoxia, via HIF1 activation, has also been identified as a key driver of PD-L1 upregulation [12]. Likewise, non-coding RNAs and RNA-binding proteins influence PD-L1 mRNA stability and translation [13]. Collectively, these molecular and microenvironmental factors influence PD-L1 expression in tumors, potentially leading to false positive cases where high PD-L1 expression does not reflect a tumor’s T cell infiltration and actual susceptibility to checkpoint inhibitors (Figure 1).

### 2.2. The Impact of PD-L1 Glycosylation on Immunohistochemical Analysis

Glycosylation plays a crucial role in PD-L1 regulation by altering protein structure and antibody recognition [14]. This can lead to false negatives in PD-L1 assessment by immunohistochemistry due to reduced antibody binding. To address this issue, Lee et al. [15] developed a deglycosylation technique that enzymatically removes glycan modifications from cell surface antigens. Their findings demonstrated that deglycosylation significantly enhanced anti-PD-L1 antibody binding, improving both signal intensity and predictive accuracy for checkpoint inhibitor response in a cohort of 44 NSCLC patients. These results underscore the importance of standardized sample processing protocols for PD-L1 assessment [16].

### 2.3. PD-L1 Expression Stability During Treatment

A key challenge in using PD-L1 as a predictive biomarker is its instability over time, particularly in response to therapy. Several studies have highlighted how the PD-L1 expression can fluctuate under different treatment conditions. For instance, chemotherapy has been shown to upregulate PD-L1 expression, with some studies reporting a 35% increase in PD-L1 levels in patients with recurrent NSCLC after platinum-based chemotherapy [17]. Likewise, targeted therapies, such as EGFR and RAF (Rapidly Accelerated Fibrosarcoma) inhibitors, not only block oncogenic pathways but can also reduce PD-L1 expression, further complicating its predictive value [2]. Additionally, antibody-dependent cell-mediated cytotoxicity (ADCC) represents a key immune mechanism through which antibody-coated tumor cells are targeted and killed by NK cells. Notably, IgG1-type therapeutic antibodies (such as pembrolizumab) can enhance IFN-γ release from NK cells, potentially increasing PD-L1 expression in tumors. This suggests that PD-L1 levels could be actively modified during treatment with checkpoint inhibitors, particularly when combined with other therapeutic antibodies [18]. Collectively, these clinical observations raise significant concerns about the validity of using a static, pre-treatment PD-L1 assessment at diagnosis to guide long-term checkpoint inhibitor therapy decisions.

## 3. Challenges for PD-L1 Expression as a Biomarker

Despite the transformative potential of checkpoint inhibition, clinical experience has revealed substantial heterogeneity in therapeutic responses. A meta-analysis reported that higher PD-L1 expression levels were associated with greater treatment effects, while a small fraction of patients exhibited responses independently of PD-L1 expression [19]. These data, obtained from a large sample of 4174 patients in eight controlled trials, suggested that PD-L1 expression alone was insufficient in identifying which patients should be offered PD-1 or PD-L1 blockade therapy. This limitation underscores the pressing need for robust predictive biomarkers to guide patient selection and therapeutic decision-making for the application of checkpoint inhibitors [20]. For patients with metastatic NSCLC, the development of antibodies against PD-L1 or PD-1 either in first-line or in second-line therapy has offered unprecedented, prolonged survival for a significant proportion of these patients [21]. In the context of monotherapy, high PD-L1 expression (tumor proportion score ≥50%) has emerged as the most clinically relevant predictive biomarker for the efficacy of anti-PD-(L)1 antibodies [21]. In contrast, in the setting of chemo-immunotherapy combinations in NSCLC, clinical trial analyses have shown that pembrolizumab provides comparable progression-free survival benefits regardless of PD-L1 expression levels (≥1% vs. <1%) [21]. These findings led both U.S. and European health regulatory agencies to approve this treatment approach regardless of PD-L1 expression status.

Moreover, the reliability of PD-L1 analysis has been called into question by studies such as that of Di Federico et al., who analyzed over 400 tumor sample pairs from NSCLC patients treated with checkpoint inhibitors [22]. Their findings revealed only moderate concordance in PD-L1 expression scores between paired samples, raising concerns about the consistency and clinical utility of PD-L1 as a decision-making tool. In fact, the utility of PD-L1 expression as a standalone predictor is constrained by several biological and methodological limitations [23]. Tumor PD-L1 expression exhibits significant spatial and temporal heterogeneity, potentially leading to sampling bias in small biopsies. Moreover, PD-L1 expression represents a dynamic phenotype that fluctuates in response to inflammatory cytokines, particularly interferon-gamma, rather than a fixed tumor characteristic. This adaptability complicates interpretations based on single time-point assessments. Technical factors further complicate PD-L1 expression measurement, including variability in antibody clones, detection platforms, scoring algorithms, and positivity thresholds across different companion diagnostic assays. This absence of standardized methodology may result in inconsistent PD-L1 assessment across clinical trials and routine practice, and have compromised inter-study comparability and hindered the establishment of universally applicable cutoff values (Figure 1).

## 4. As PD-L1 Expression Is Limited in Its Predictive Value, What Should We Propose Next?

Regarding tumoral PD-L1 expression and, more particularly, in the context of immunotherapy for NSCLC, should we throw out the baby with bath water? Certainly not, and for several reasons. One of the main ones is that there are no truly faithful alternatives for predicting CPI efficacy, particularly for NSCLC patients. It is clear, for instance, that the tumor mutational burden (TMB), which quantifies the number of somatic mutations per megabase of DNA, has emerged as a promising complementary biomarker [24]. A high TMB correlates with increased neoantigen production, potentially eliciting stronger anti-tumor immune responses upon checkpoint blockade. Similarly, microsatellite instability (MSI) and deficient mismatch repair (dMMR) status have demonstrated value in identifying hypermutated tumors likely to respond to immunotherapy [25]. Although the TMB and neoantigen profiling have been explored, meta-analyses suggest that their correlation with the checkpoint inhibitor response remains inconsistent [26]. This may be due to the complex, individualized nature of antigen presentation and T cell recognition [27,28].

Immune cell infiltration patterns, particularly the density and spatial distribution of CD8+ T cells within the tumor microenvironment, provide critical information regarding the pre-existing immune response [29]. Multiplex immunohistochemistry and spatial transcriptomics enable a comprehensive characterization of the immune contexture, offering insights into the complex cellular interactions that shape immunotherapy outcomes [30]. Gene expression signatures capturing interferon signaling, T cell function, and inflammatory pathways have also shown promise in predicting ICI efficacy [31]. These transcriptomic profiles may more accurately reflect the functional state of the tumor immune microenvironment compared to static assessments of PD-L1 protein expression.

Emerging peripheral blood-based biomarkers such as soluble PD-L1 in exosomes have shown promise as prognostic and predictive markers in checkpoint inhibitor-treated NSCLC patients, supporting the role of liquid biopsy approaches [32,33]. Additionally, advancements in artificial intelligence (AI) for biomarker discovery have led to AI-driven models that integrate PD-L1 expression with multimodal data, including genomics, radiomics, and real-world patient data (Figure 1). These PD-L1-enriched meta-biomarkers may enhance precision medicine strategies in immuno-oncology [34].

## 5. Conclusions and Perspectives

Despite its limitations, it is clear that the mere determination of PD-L1 remains a valuable but imperfect biomarker for predicting CPI response, particularly in NSCLC. Its main limitations stem from the complexity of its regulation, which is influenced by both immune-mediated IFN-γ signaling and oncogenic pathways independent of T cell interactions. Additionally, PD-L1 expression is dynamic, changing over time and under different therapeutic pressures, further complicating the predictive reliability of a static snapshot.

Future advances in multi-omics biomarker development—combining PD-L1 expression with genetic, proteomic, and radiomic data—may improve patient selection for checkpoint inhibitors. Large-scale prospective validation studies will be crucial in refining these predictive models. Additionally, machine learning algorithms, such as SCORPIO, which incorporate routine blood tests and clinical characteristics, may provide more accurate and dynamic assessments of CPI efficacy [35]. As precision oncology evolves, the role of PD-L1 in predictive biomarker strategies is likely to shift from a standalone marker to an integrated component of AI-driven multi-omics platforms, ultimately improving patient stratification and treatment outcomes.

## Data Availability

Not applicable. There are no data or materials involved in this review.
